# Functional Characterization of Allatostatin C (PISCF/AST) and Juvenile Hormone Acid O-Methyltransferase in *Dendroctonus armandi*

**DOI:** 10.3390/ijms23052749

**Published:** 2022-03-02

**Authors:** Yaya Sun, Danyang Fu, Bin Liu, Linjun Wang, Hui Chen

**Affiliations:** 1 State Key Laboratory for Conservation and Utilization of Subtropical Agro-Bioresources, College of Forestry and Landscape Architecture, South China Agricultural University, Guangzhou 510642, China; sunyaya@nwafu.edu.cn; 2 College of Forestry, Northwest A&F University, No. 3 Taicheng Road, Yangling, Xianyang 712100, China; fudanyang@nwsuaf.edu.cn (D.F.); liubin95@nwsuaf.edu.cn (B.L.); 2020060253@nwafu.edu.cn (L.W.)

**Keywords:** Chinese white pine beetle, juvenile hormone (JH), development, frontalin, RNAi

## Abstract

Allatostatin C (PISCF/AST) is a neuropeptide gene that affects juvenile hormone (JH) synthesis in the corpora allata. Juvenile hormone acid O-methyltransferase (*JHAMT*) is a key gene in the JH biosynthetic pathway. In this study, two genes encoding *DaAST* and *DaJHAMT* were cloned. Both *DaAST* and *DaJHAMT* were expressed in the larvae, pupae and adults of Chinese white pine beetle (*Dendroctonus armandi*), and highly expressed in the head and the gut. The expression of the two genes was induced by JH analog (JHA) methoprene and the functions of the two genes were then investigated by RNAi. Considering the role of hormones in metamorphosis, JHA significantly induced *DaAST* and *DaJHAMT* in the larval stage. *DaAST* knockdown in larvae, pupae and adults significantly increased the *DaJHAMT* mRNA levels. Moreover, knockdown of *DaAST* instead of *DaJHAMT* increased pupae mortality and the abnormal rate of emergence morphology and reduced emergence rates. However, knockdown of *DaJHAMT* instead of *DaAST* significantly reduced frontalin biosynthesis in adult males. The results showed that *DaAST* acts as an allatostatin and inhibits JH biosynthesis, and that *JHAMT* is a key regulatory enzyme for JH synthesis in the *D. armandi*.

## 1. Introduction

Insect juvenile hormone (JH), a multifunctional hormone secreted from the corpora allata (CA), plays a role in multiple physiological events including diapause, pheromone production, polyphenisms, growth, molting, metamorphosis and reproduction [1,2,3,4,5,6,7]. The JH biosynthetic pathway was conventionally divided into two phases: early steps (the upstream mevalonate pathway, MVAP) and late steps (the isoprene branch pathway, JH-branch) [8,9]. The early steps follow the mevalonic acid pathway to form farnesyl pyrophosphate (FPP) and involve several enzymes. Initially, three units of Acetyl-CoA are condensed into mevalonate through three sequential steps involving acetoacetyl-CoA thiolase (*AACT*), 3-hydroxy-3-methylglutaryl-CoA synthase (*HMG-S*) and 3-Hydroxy-3-methylglutaryl-CoA reductase (*HMG-R*). Then, mevalonate is converted to isopentenyl diphosphate (IPP) through three enzymatic reactions catalyzed by mevalonate kinase (*MK*), phosphomevalonate kinase (*PMK*), and mevalonate diphosphate decarboxylase (*MPDC*) [10]. Isopentenyl diphosphate isomerase (*IPPI*) catalyzes the conversion of isopentenyl pyrophosphate (IPP) to dimethylallyl pyrophosphate (DMAPP); subsequently, IPP and DMAPP were condensed head-to-tail manner to produce geranyl diphosphate (GPP); this head-to-tail condensation can be repeated by the further reaction of GPP with IPP, yielding the JH precursor farnesyl diphosphate (FPP) [11]. The expressions of eight enzymes of the mevalonate pathway have been previously examined in *Dendroctonus armandi* [12,13]. During the late steps proceeding from farnesyl diphosphate to JH, FPP is transformed sequentially to farnesol, farnesal, farnesoic acid, methyl farneosate and JH [10,14,15,16]. The juvenile hormone acid O-methyltransferase (*JHAMT*) is a key regulatory enzyme in the isoprene branch pathway that is essential for insect growth and reproduction [17,18]. For example, silencing the *TcJHAMT* gene in the larval stage of *Tribolium castaneum* resulted in premature larva-pupa metamorphosis [19]. In particular, the genes encoding enzymes for the mevalonate pathway and the last two steps of the JH branch pathway (farnesoic acid (FA) epoxidase and *JHAMT*) have been identified in multiple species [10,17,18,19,20,21,22,23,24].

Different factors are involved in the regulation of JH synthesis [14]. These include allatoregulatory neuropeptides, biogenic amines [25,26], nutritional signals [27] and nuclear receptors [28]. In insects, one of the largest groups of neuropeptides is the group of allatostatins (*ASTs*). *ASTs* have been identified in beetles, cockroaches, crickets, flies, moths, stick insects, and termites [29,30,31,32,33,34,35,36,37]. *ASTs* were first divided into three distinct families: Type A [37], Type B [33] and Type C allatostatin [29,38,39]. However, because the classification of families did not address either the biological properties of these peptides or the structure of the amino acid chains of these peptides, Coast & Schooley (2011) systematized the nomenclature of these neuropeptides [40]. They proposed that A Type allatostatin should be named FGL/AST because of the presence of an FGL sequence at the C-terminal end, Type B should be named MIP/AST because of the myoinhibitory properties of these peptides on the visceral muscles, and Type C should be named PISCF/AST, because there is an uncommon PISCF-OH sequence at the C-terminal end [40]. In coleopteran species, in vitro studies showed that the *Tribolium castaneum AS-B3* peptide inhibited CA activity, while *TcAS-C* inhibited or stimulated JH biosynthesis [41]. Furthermore, the injection of double-stranded RNAs (dsRNAs) targeting *TcAS-C* and *TcAS-B* into young *T. castaneum* pupae prolonged pupae duration and resulted in the production of approximately 50% of deformed adults. The other 50% were intact in shape [42]. Moreover, in *Leptinotarsa decemlineata*, the mRNA levels of *LdJHAMT* were significantly increased and the larval growth and delayed development of *L. decemlineata* were significantly affected after continuous ingestion of *dsLdAS-C* [43]. However, the in vivo allophilic activity of *AST-C* peptides in other *Discoptera* species remains unproven.

RNA interference (RNAi) is the process by which exogenous double-stranded RNA (dsRNA) silences complementary endogenous messenger RNA (mRNA) [44]. Since being discovered in *Caenorhabditis elegans* [45], RNAi first achieved successful insect action in *Drosophila melanogaster* [46]. Due to its high specificity, RNAi has great potential for pest control [47,48,49]. It has rapidly developed into a widely used tool in a variety of insects, including Hymenoptera [50,51], Diptera [52,53], Coleoptera [54,55] and Lepidoptera [56,57,58]. There are two ways to deliver dsRNA to insect target tissues: injection and ingestion [59,60]. Of these two methods, the RNAi feeding program has several advantages over the injection method, the most important of which is that it is more convenient and labor-saving. It has been reported that transgenic plants that produce dsRNA targeting selected insect genes have shown inhibitory effects on cotton bollworm [61] and western corn rootworm [47]. Their expression induced by JH analog (JHA) was analyzed in different tissues at different developmental stages through a series of RNAi experiments.

In this study, we used RNA interference to characterize the functions of the two genes *DaAST* and *DaJHAMT* in the Chinese white pine beetle (*Dendroctonus armandi*), and analyzed the expression levels of JH biosynthesis genes after knocking down *DaAST* and *DaJHAMT*; their effects on development were also investigated. More broadly, the injection of bacterially expressed dsRNA may be suitable for large-scale optimized gene screening of RNAi-ingestible plants to provide a theoretical basis for understanding the molecular mechanisms of allatostatin C (PISCF/AST) and juvenile hormone acid O-methyltransferase in Chinese white pine beetle.

## 2. Results

### 2.1. Identification of DaAST and DaJHAMT Genes

**Figure 1:**
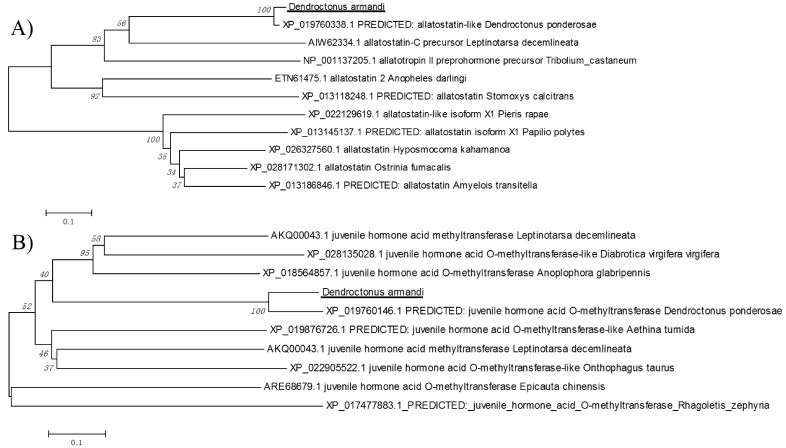
Phylogenetic tree of allatostatin (*DaAST*) and juvenile hormone acid O-methyltransferase (*DaJHAMT*) from *Dendroctonus armandi*. (**A**) Phylogenetic analysis of *DaAST*; (**B**) Phylogenetic analysis of *DaJHAMT*. The two sequences from *D. armandi* are marked in black. The phylogenetic trees were constructed with MEGA7.0 using the neighbor-joining method. Values indicated at the nodes are bootstrap values based on 1000 replicates.

*DaAST* was identified in *D. armandi* and the full-length sequences shared high identity (64–69%) with *Dendroctonus ponderosae*, *T. castaneum* and *L. decemlineata* (Table 1). The deduced amino acid sequence of *DaJHAMT* had high identity (53%) to the *JHAMT* of *D. ponderosae*. *DaJHAMT* also showed a relatively high amino acid identity (36.8% and 36.5%) to the *JHAMT* predicted from the genomes of *Nicrophorus vespilloides* and *Aethina tumida*. These sequences of *D. armandi* had the highest identity to *D. ponderosae* according to a neighbor-joining method analysis of the putative full-length amino acid sequences (Figure 1).

### 2.2. Physicochemical Properties and Bioinformatic Analysis

The full-length open reading frames (ORFs) of *DaAST* and *DaJHAMT* were 345 bp (*DaAST*) and 848 bp (*DaJHAMT*) encoding 114 (*DaAST*) and 275 (*DaJHAMT*) amino acids. Respectively, the predicted molecular masses were 13.28 kDa (*DaAST*) and 15.11 kDa (*DaJHAMT*), and the isoelectric point ranged from 8.66 (*DaAST*) to 9.04 (*DaJHAMT*); the predicted subcellular location of *DaAST* and *DaJHAMT* by Target P1.1 program suggests cytoplasmic location (Table 2). 

**Figure 2:**
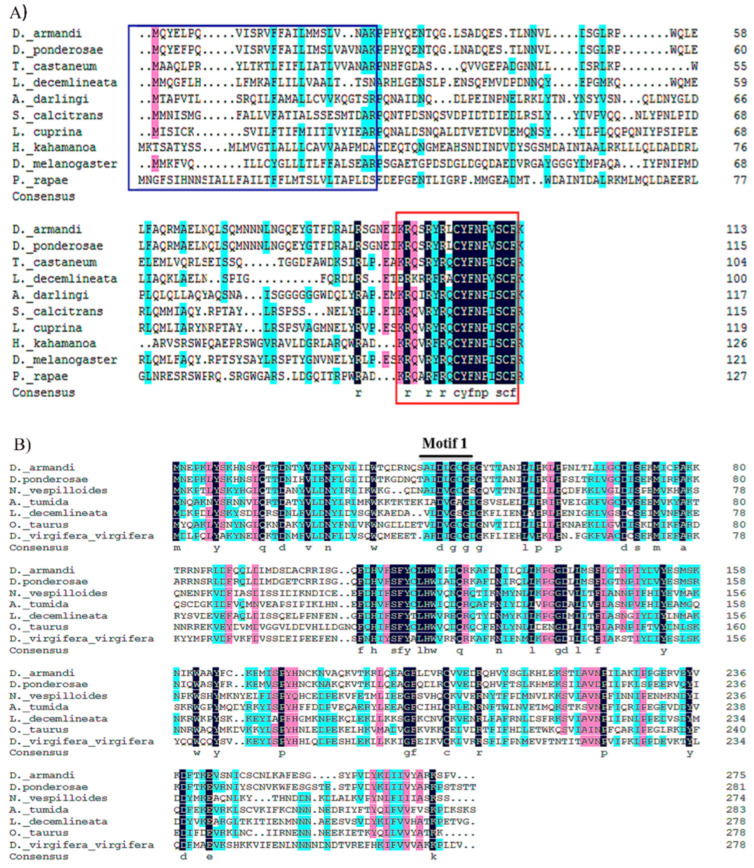
Structure of *DaAST* and *DaJHAMT* in *D. armandi*. (**A**) Alignment of putative *DaAST* sequence in beetle species and their consensus sequences with *D. armandi* sequences identified in this study. The blue color indicates similarities in the amino acid sequence. Blue frames: signal peptides at the N-termini; black: endopeptidase cleavage sites; red frames: other deduced MIP isoforms. (**B**) Alignment of *DaJHAMT* sequences. The conserved motif I of all SAM-dependent methyl transferases is marked with a bar above the sequences.

According to the expected cleavages from insect neuropeptide convertase, the archetypal *AST*-C peptides in hymenopteran, anopluran, hemipteran and zooplankton species have the primary sequence SYWKQCAFNAVSCF amide [65]. The PISCF/AST sequences of *L. decemlineata*, *D. ponderosae* and *D. armandi* are RFRA (L) CYFNPVSCF (Figure 2A) [66]. It is known that the putative SAM-binding motif (motif I) is well conserved in all five methyl transferases, hh (D/E) hGXGXG (where h represents a hydrophobic residue). The motif I of *DaJHAMT* (ALDLGCGEG) was nearly identical to that of *DponJHAMT* (AIDLGCGEG) (Figure 2B). The *DaAST* and *DaJHAMT* sequences are structurally similar to those of *D. melanogaster* and *Tenebrio molitor,* further confirming that they are homologues of *AST* and *JHAMT*.

### 2.3. DaAST and DaJHAMT Transcript Levels in D. armandi at Different Life Stages and in Different Tissues

**Figure 3:**
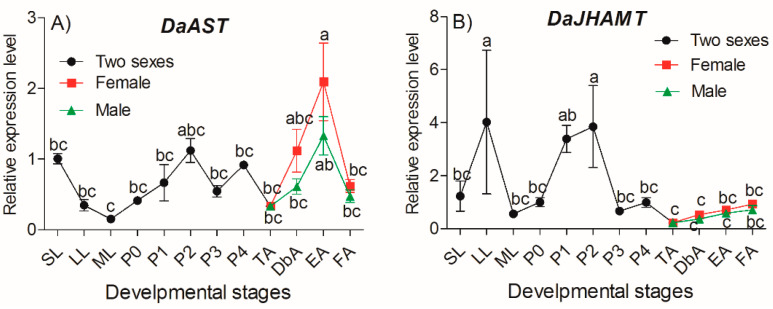
Relative expression of *DaAST and DaJHAMT* genes in different stages of *D. armandi*. (**A**) *DaAST*; (**B**) *DaJHAMT.* Small larvae (SL), large larva (LL), mature larvae (ML), pupae (P0, P1, P2, P3, P4), teneral adults (TA), dark brown adults (DbA), emergent adults (EA) and feeding adults (FA) of *D. armandi*. The results represent the mean ± SE of three independent experiments. All templates were normalized with *CYP4G55* and *β-actin*. The 2^−ΔΔCt^ and SE values were used for plotting and the significant differences between different stages of *DaAST or DaJHAMT* were marked with letters (one-way ANOVA, *p* < 0.05, with Tukey’s test of multiple comparisons).

The transcript levels of *DaAST* throughout the developmental stages were determined using qRT-PCR. Relative to larvae, one-way ANOVA showed statistically significant differences in transcript expression levels among different development stages (*F =* 6.128, df = 14, *p* < 0.0001) (Figure 3). The expression of *DaAST* showed a downward trend in larvae, beginning with small larvae and reaching the lowest value in mature larvae. The expression then decreased in the pupae and reached a peak on the second day of the pupal stage, then dropped to a second trough that appeared at the teneral stage. In adults, the expression reached a maximum in emergent females and decreased to some extent, beginning in feeding females. The expression levels in females were higher than those in males (Figure 3A).

The transcript levels of *DaJHAMT* throughout the developmental stages were determined using qRT-PCR. Relative to larvae, a one-way ANOVA showed statistically significant differences in transcript expression levels among different development stages (*F =* 2.402, df = 14, *p =* 0.022) (Figure 3B). In larvae, the expression of *DaJHAMT* first increased and then decreased. From pupae to the teneral stage, the expression patterns of *DaJHAMT* were similar to those of *AST*. In adults, the expression levels of *DaJHAMT* remained constant from the teneral stage to the feeding adult stage but were lower than the levels in pupae (Figure 3B).

The tissue distribution of *DaAST* and *DaJHAMT* transcripts in various tissues from three developmental stages (larva, pupa, adult) were investigated by real-time RT-PCR analysis.

**Figure 4:**
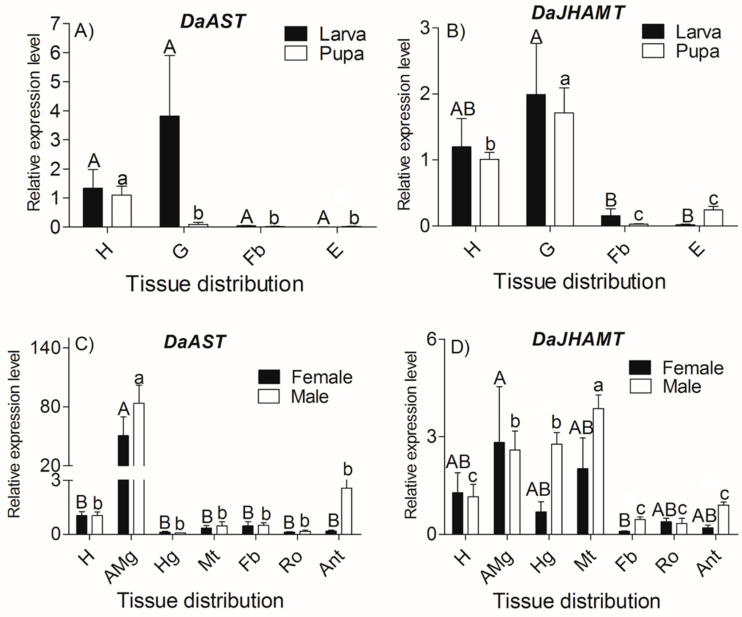
Relative expression of *DaAST and DaJHAMT* genes (mean ± SE) in different tissues of larvae, pupae and emergent adults. (**A**) *DaAST* of larvae and pupae; (**B**) *DaJHAMT* of larvae and pupae; (**C**) *DaAST* of emergent adults and (**D**) *DaJHAMT* of emergent adults. The tissues included the head (H), anterior midgut (AMg), hindgut (Hg), Malpighian tubule (Mt), fat body (Fb), reproductive organ (Ro: testes of males and ovaries of females), antennae (Ant), gut (G) and epidermis (E) of *D. armandi*. The results represent the means ± SE of three independent experiments. In both graphs, transcript levels are represented as relative expression to the head (H) of males and females. All templates were normalized with *CYP4G55* and *β-actin*. The 2^−ΔΔCt^ and SE values were used for plotting. Uppercase letters mark significant differences in females or larvae, while lowercase letters mark males or pupae (one-way ANOVA, *p* < 0.05, with Tukey’s test of multiple comparisons).

For *DaAST*, at the larva stage, statistically significant differences were not found among different tissues of *DaAST* (Figure 4A); in the pupa, the expression of *DaAST* in the head reaches the highest level (*F =* 11.562, df = 3, *p =* 0.003); During the period of adult emergence, the highest expression level of *DaAST* was observed in female and male anterior midguts (female: *F =* 7.038, df = 6, *p =* 0.001; male: *F =* 19.926, df = 6, *p* < 0.0001) (Figure 4C). 

For *DaJHAMT*, quantitative RT-PCR (qRT-PCR) analysis of *DaJHAMT* showed that statistically significant differences were found among different tissues in all developmental stages. *DaJHAMT* was highly expressed in larval and pupal heads, and the highest expression was found in the larval and pupal guts (larva: *F =* 4.386, df = 3, *p =* 0.042; pupa: *F =* 15.022, df = 3, *p =* 0.001) (Figure 4B). During the period of adult emergence, statistically significant differences were not found among the different tissues of females (*F =* 1.703, df = 6, *p =* 0.193). Meanwhile, the highest expression level of *DaJHAMT* was observed in male Malpighian tubules (*F =* 15.369, df = 6, *p* < 0.0001); the anterior midgut and hindgut had higher expression volumes than the other tissues (Figure 4D). 

### 2.4. Effects of JHA Injection on Transcript Levels of DaAST and DaJHAMT

**Figure 5:**
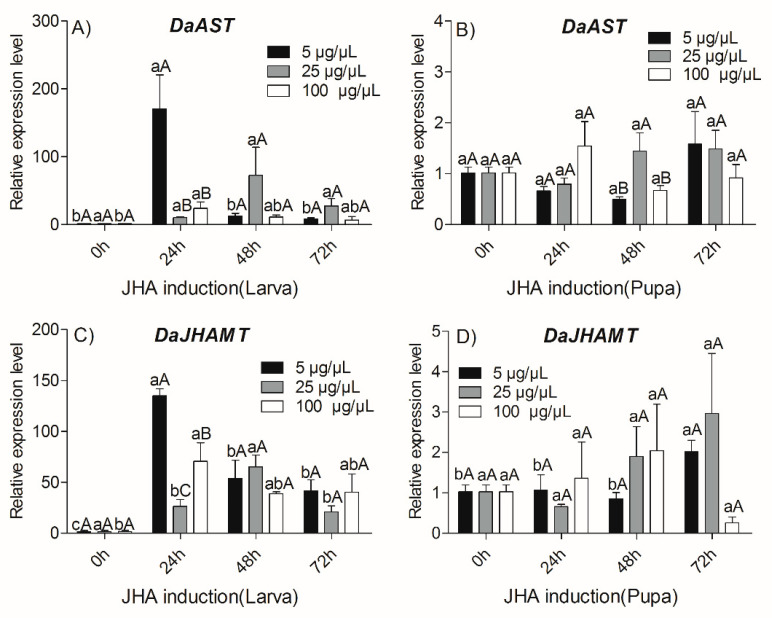
Relative expression of *DaAST and DaJHAMT* genes with JHA (5, 25, 100 μg/μL) at different times in larvae and pupae. (**A**) *DaAST* mRNA level in larvae; (**B**) *DaAST* mRNA level in pupae; (**C**) *DaJHAMT* mRNA level in larvae and (**D**) *DaJHAMT* mRNA level in pupae. The results represent the means ± SE of three independent experiments. In all graphs, the transcript levels are shown relative to acetone-treated (control) larvae and pupae. All templates were normalized with *CYP4G55* and *β-actin*. The 2^−ΔΔCt^ and SE values were used for plotting. Different letters indicate significant differences at *p* < 0.05 (two-way ANOVA). Uppercase letters mark significant differences between concentrations at the same time, while lowercase letters mark the significant differences between times at the same concentration.

To reveal the molecular mechanism of the influence of JHA on the expression of *DaAST* and *DaJHAMT*, the relative expression profiles of *DaAST* and *DaJHAMT* were analyzed by qRT-PCR at several time points after JHA treatment. We first examined the effects of JHA application in larvae. The qRT-PCR analysis showed that both doses (5 and 100 µg/µL of methoprene) of JHA application significantly influenced the expression of *DaAST* and *DaJHAMT* (*p* < 0.05) (Figure 5). JHA treatments increased the *DaAST* and *DaJHAMT* expressions from 0 to 24 h at both doses (5 µg/µL and 100 µg/µL), then increased expression from 24 to 72 h post treatment (Figure 5A,C). The expression of *DaJHAMT* was upregulated from 0 to 48 h after treatment (25 µg/µL), compared with the larvae controls. The expression of *DaAST* and *DaJHAMT* between larvae treated with different doses only differed significantly at 24 h post treatment. Next, we examined the effects of JHA application on pupae. Compared with the pupae of the control group, there was no significant difference in the expression of *DaAST* at different times at all doses (Figure 5B). The expression of *DaJHAMT* in the 5 µg/µL treatment group increased with time until 72 h post treatment, however, there was no significant difference over time for other doses (Figure 5D). The expression levels of *DaAST* and *DaJHAMT* between the pupae treated with different doses were only significantly different at 48 h post treatment, and pupae at a dose of 25 µg/µL had the highest expression levels compared with those treated with 5 µg/µL and 100 µg/µL.

### 2.5. RNAi Effect of DaAST and DaJHAMT

#### 2.5.1. Determination of *DaAST* and *DaJHAMT* Silencing by qRT-PCR

**Figure 6:**
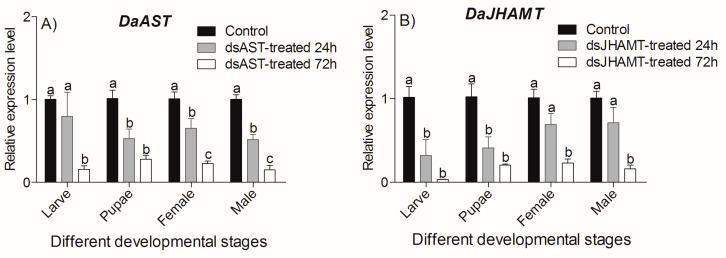
The qRT-PCR analysis of *DaAST and DaJHAMT* gene transcript patterns in *D. armandi* 24 and 72 h after dsRNA injection. (**A**) The qRT-PCR analysis of *DaAST* transcript pattern; (**B**) the qRT-PCR analysis of *DaJHAMT*. The standard errors of the means of three biological replicates are represented by error bars. Different letters above the bars indicate a significant difference (One-way ANOVA, *p* < 0.05, with Tukey’s test of multiple comparisons).

Analysis of the expression of *DaAST* and *DaJHAMT* after injection with simultaneous dsAST and dsJHAMT confirmed that both genes were successfully knocked down at all stages (Figure 6). For the *DaAST* and *DaJHAMT* expression level analysis, compared with the negative control, the transcription levels of *DaAST* and *DaJHAMT* measured by qRT-PCR at 72 h were significantly lower than those at 24 h (*p* < 0.05). These results indicate that RNAi through injection and smearing requires the accumulation of dsRNA in the beetles, and that continuous feeding can inhibit gene expression. These results indicate that *DaAST* and *DaJHAMT* gene silencing can reduce the expression of the target genes.

#### 2.5.2. Knockdown Effect of Injecting dsAST and dsJHAMT Separately

**Figure 7:**
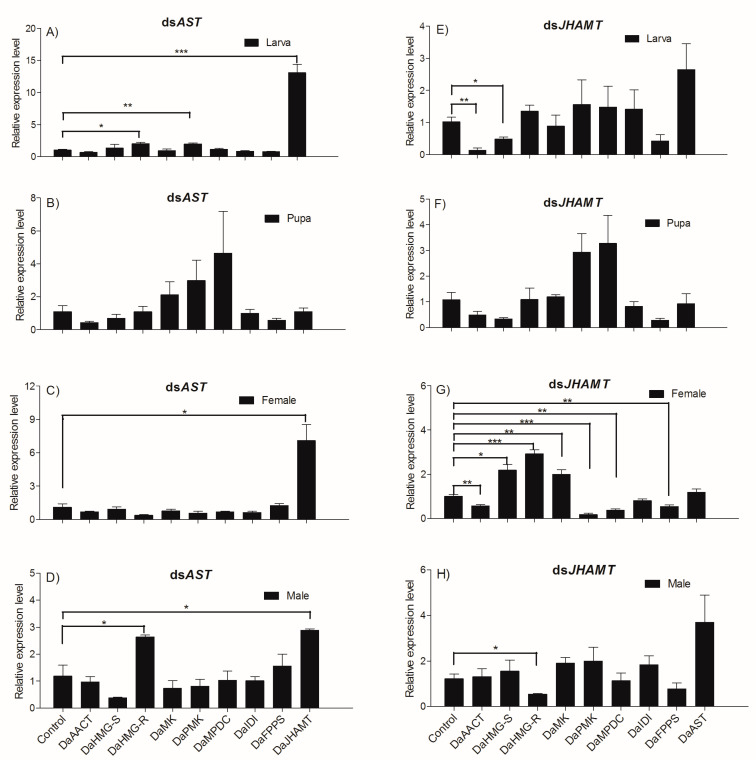
Knockdown of *DaAST* and *DaJHAMT* affected JH biosynthesis and the mevalonate pathway. The larvae, pupae and emergent adults were allowed to ingest DEPC treated water (negative control), dsAST or dsJHAMT for three days. The expression levels of *DaAST*, *DaJHAMT* and mevalonate enzyme-encoding genes were measured. (**A**) Knocked down *DaAST* in larvae, (**B**) knocked down *DaAST* in pupae, (**C**) knocked down *DaAST* in female adults, (**D**) knocked down *DaAST* in male adults, (**E**) knocked down *DaJHAMT* in larvae, (**F**) knocked down *DaJHAMT* in pupae, (**G**) knocked down *DaJHAMT* in female adults and (**H**) knocked down *DaJHAMT* in male adults. The columns represent averages with vertical lines indicating SE. Asterisks denote significant differences (unpaired *t*-test; * *p* < 0.05, ** *p* ≤ 0.01, *** *p* ≤ 0.001).

In this study, we found that knockdown of *DaAST* in larvae enhanced the expression of *DaHMG-R*, *DaPMK* and *DaJHAMT* (*DaHMG-R*: t *=* −4.191, df = 4, *p =* 0.014; *DaPMK*: t *=* −5.459, df = 4, *p =* 0.005; *DaJHAMT*: t *=* −9.118, df = 4, *p =* 0.001) (Figure 7A), with no significant expression of MVA pathway genes when knocking down *DaAST* in pupae (Figure 7B). Knockdown of *DaAST* in females enhanced the expression of *DaJHAMT* (*DaJHAMT*: t *=* −4.035, df = 4, *p =* 0.016) (Figure 7C), whereas knockdown of *DaAST* in males enhanced the expression of *DaHMGR* and *DaJHAMT* (*DaHMG-R*: t *=* −3.574, df = 4, *p =* 0.023; *DaJHAMT*: t *=* −4.232, df = 4, *p =* 0.013) (Figure 7D).

We found that *DaJHAMT* deletion in larvae significantly downregulated the expression of the MVA pathway gene *DaAACT* and *DaHMG-R* (*DaAACT*: t *=* 5.406, df = 4, *p =* 0.006; *DaHMG-R*: t *=* 3.407, df = 4, *p =* 0.027) (Figure 7E). We found that knockdown of *DaJHAMT* in females significantly downregulated the expression of *DaAACT, DaPMK, DaMPDC* and *DaFPPS* (*DaAACT*: t *=* 5.134, df = 4, *p =* 0.007; *DaPMK*: t *=* 8.983, df = 4, *p =* 0.001; *DaMPDC*: t *=* 6.999, df = 4, *p =* 0.002; *DaFPPS*: t *=* 4.921, df = 4, *p =* 0.008) (Figure 7F, but significantly upregulated the expression of *DaHMG-S, DaHMG-R* and *DaMK* (*DaHMG-S*: t *=* −4.416, df = 4, *p =* 0.012; *DaHMG-R*: t = −10.053, df = 4, *p =* 0.001; *DaMK*: t *=* −4.682, df = 4, *p =* 0.009) (Figure 7G). Knockdown of *DaJHAMT* in males significantly downregulated the expression of *HMGR* (*DaHMG-R*: t *=* 3.211, df = 4, *p =* 0.033) (Figure 7H).

#### 2.5.3. The Effects of RNAi of dsAST and dsJHAMT on the Development of *D. armandi*

**Figure 8:**
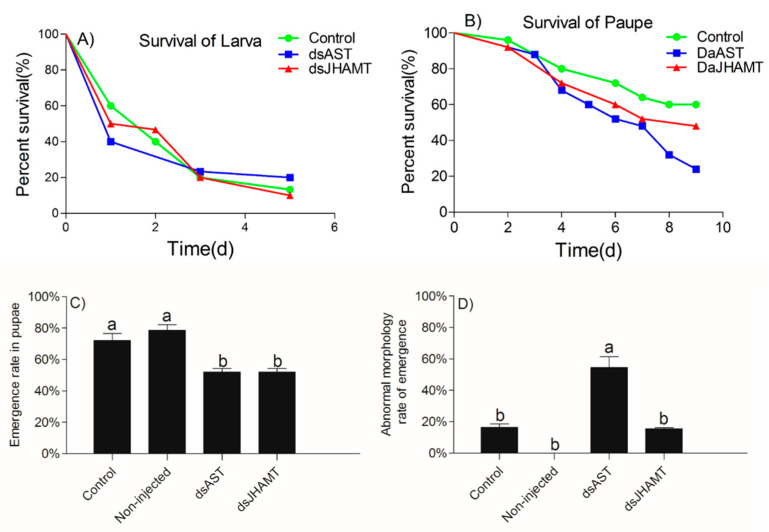
Survival emergence and abnormal morphology rate in *D. armandi* larvae and pupae after RNAi. Effect of bacterially expressed dsAST/JHAMT on *D. armandi* larvae and pupae. (**A**) Survival rate in larvae, (**B**) survival rate in pupae, (**C**) emergence rate in pupae and (**D**) abnormal morphology rate in pupae. There were 25 samples in each biological repetition. The data were analyzed by a one-way ANOVA test. Different letters above the bars indicate a significant difference (*p* < 0.05).

After the larvae were treated with engineered bacteria, the survival rates of the control (vector control), dsAST and dsJHAMT groups were 13.33%, 20.00% and 10.00%, respectively, on Day five (Figure 8A). The Kaplan–Meier method (log rank (Mantel–Cox)) was used to analyze the survival rates. There was no significant difference in the survival rates of the dsAST and dsJHAMT groups compared with the control group (dsAST: χ^2^ = 0.037, df = 1, *p* = 0.847; dsJHAMT: χ^2^ = 0.010, df = 1, *p* = 0.919). After the pupae were treated with engineered bacteria, the survival rates of the control (vector control), dsAST and dsJHAMT groups on Day nine was 60.00%, 24.00%, and 48.00%, respectively. (Figure 8B). Survival in the dsAST group was 36% lower than in the control group (χ^2^ = 5.083, df = 1, *p* = 0.024) whereas there was no difference in survival between the dsJHAMT and control groups (χ^2^ = 0.651, df = 1, *p* = 0.52). In addition to the larval and pupal survival rate, the effects of dsAST and dsJHAMT on the emergence rate of pupae and the rate of abnormal morphology in emergent adults were also analyzed. The emergence rates of the dsAST and dsJHAMT groups were both 52.00%. These rates were significantly lower than the emergence rate of the control (vector control), which was 72.00% (*F =* 17.0, df = 3, *p =* 0.001) (Figure 8C). The abnormal morphology rates in emergent adults of the control (vector control) and dsJHAMT groups were 16.39% and 15.45%, respectively. These rates were significantly lower than that of the dsAST groups, which was 54.46% (*F =* 38.2, df = 3, *p <* 0.001) (Figure 8D). Taken together, these data show that *DaAST* and *DaJHAMT* gene silencing was able to disturb the growth and development of *D. armandi,* and that dsAST had the greatest interference effect.

#### 2.5.4. Adult Development of the dsRNA Phenotypes

**Figure 9:**
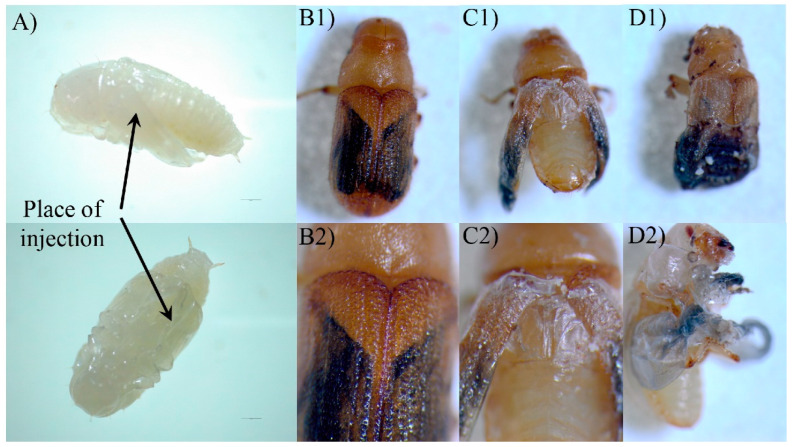
Biological morphology of *D. armandi* from the pupa to adult stage. (**A**) Arrows point to the place of pupa injection; (**B1**,**B2**) normal adult morphology in the control group; (**C1**,**C2**) abnormal morphology of adult *D. armandi* that were treated with dsAST; (**D1**,**D2**) abnormal morphology of adult *D. armandi* after dsJHAMT treatment.

Figure 9 shows the phenotypes of *D. armandi* adults produced by dsRNA-mediated transcript silencing. The injection of pupae with dsRNA for *DaAST* resulted in shape abnormalities in 54.46% of the treated beetles. Many parts of their appendices, including the wings and parts of the legs, were deformed or partly covered with old epicuticle. The abnormal morphology rate in emergent adults in the control group (vector control) was significantly lower by 38.07% than in those of the dsAST groups, and the abnormal adults died within 4 days after emergence. The beetles with intact cuticles did not differ in shape from the controls. Moreover, the *DaAST* dsRNA-treated beetles had smaller heads and bodies, but the elytra were shorter only in deformed beetles (Table 3). 

Injection of dsRNA for *DaJHAMT* into pupae of *D. armandi* resulted in the production of about 15.45% deformed adults (Figure 9). Although there was no difference in the rate of adult emergence between intact and deformed beetles, the misshaped beetles died within two days of adult life. The elytra of the deformed beetles were much shorter in length compared with the controls and were neither tanned nor sclerotized (Figure 9). 

**Figure 10:**
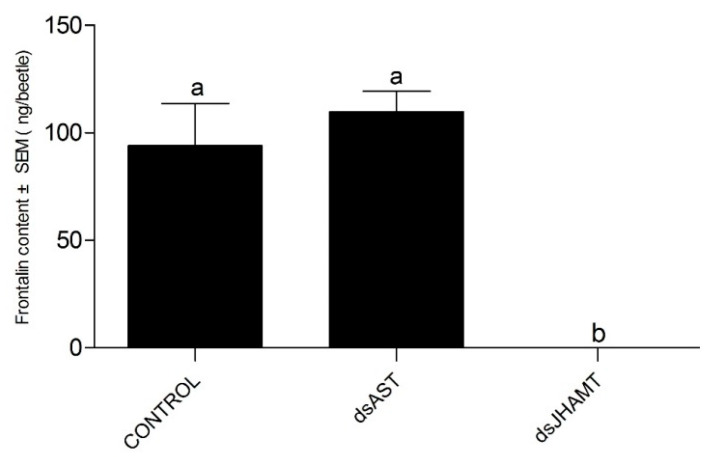
Knockdown of *DaAST* and *DaJHAMT* affected frontalin biosynthesis. Frontalin levels in male adults injected with dsRNA for the *DaAST* and *DaJHAMT* genes. Different letters above the bars indicate a significant difference (one-way ANOVA, *p* < 0.05, with Tukey’s test for multiple comparisons).

The frontalin content of adult males differed significantly among treatment groups (one-way ANOVA: *F* = 21.877, df = 2, *p* = 0.002) (Figure 10). Injection of *DaJHAMT* dsRNA significantly reduced the frontalin content to zero. However, injection of *DaAST* dsRNA had no significant effect on the amount of frontalin produced by male adults.

## 3. Discussion

In this study, allatostatin (*AST*) and juvenile hormone acid O-methyltransferase (*JHAMT*) genes identified in *D. armandi* were analyzed for their expression and function. The PISCF gene encodes a biological peptide that appears to be highly conserved compared with other heterologous proteins. For example, the peptide identified in *T. castaneum* (Trica-PISCF) also seems to be identical to the *Tenebrio molitor* sequence (Tenmo-PISCF) [43,66]. *DaJHAMT* displays the characteristic sequence of a SAM-dependent methyltransferase, which shows the structure of the active site for binding SAM and transferring the methyl group to FA or JHA [67]. 

*DaAST* was highly expressed after each molt in the pupal stage and during adult migration, while *DaJHAMT* was highly expressed on the third day of the larval stage. Because of their role in regulating JH biosynthesis, food uptake and visceral muscle contraction, *AST* peptides are also known as “brain–gut peptides” [68]. Consistent with its function, *DaAST* is usually mainly expressed in the brain. Real-time PCR showed that *DaAST* was abundantly expressed in the brains of pupae; lower expression values were found in the heads of larvae and adults, but increased expression was found in the intestines of larvae and adults. Similarly, *ASTC* of *L. decemlineata* and *T. castaneum* were also predominantly distributed in the brain + CC/CA complex and intestines [42,43].

This study found that after the application of JHA, the mRNA levels of several key genes involved in JH biosynthesis in insects, such as *AST* and *JHAMT*, were regulated by JH biosynthesis in insects after JHA administration. *JHAMT* is the most important regulator of the JH biosynthesis process [69]. The qRT-PCR results showed that the application of low concentrations of JHA stimulated the expression of *DaAST* in *D. armandi* larvae but suppressed the expression of *DaJHAMT* in *D. armandi* pupae. Meanwhile, low concentrations of JHA stimulated *DaJHAMT* expression in *D. armandi* larvae but suppressed *DaJHAMT* expression in pupae, which is consistent with observations in *Galeruca daurica* [70] and *Colaphellus bowringi* [71]. Thus, a negative feedback loop appears to control the relation between hemolymph JH titers and *AST* in *Diploptera punctata*: a higher JH titer in hemolymph stimulates *AST* gene expression, and then functional AS peptides inhibit JH biosynthesis and release in CA [72]. JH negatively feeds back to regulate the endocrine glands, which induce their products themselves. The direct or indirect inhibition of JH biosynthesis by CA first appeared in studies evaluating the effects of JH analogs. Topical application of such analogs inhibited JH biosynthesis in a dose-dependent manner, as did topical treatment with JH itself [72]. These results indicated that exogenous JH analogs paracrine release of *AST* in intact calcium and act via the hemolymph in unconnected calcium to inhibit JH biosynthesis in the brain [73,74]. 

RNAi was introduced to assess the possible effects of *DaAST* and *DaJHAMT* on JH biosynthesis in *D. armandi*. After injection with dsAST or dsJHAMT, the expression of the target gene dsAST/dsJHAMT was knocked down in larvae, pupae and adults, indicating the success of RNAi. When *D. armandi* larvae and adults ingested ds*DaAST*, the mRNA level of *DaJHAMT* increased significantly. In addition, when ds*DaAST* was knocked down in pupae, there was no significant difference in the expression of mevalonate pathway related genes. This result indicates that *DaAST* directly or indirectly affects the activity or expression of *DaJHAMT* [43,75,76], thereby inhibiting JH biosynthesis in insects [77]. This is consistent with the findings in *Spodoptera frugiperda* and *Clostera anastomosis* [78,79].

The injection of dsRNA into freshly molted *D. armandi* pupae resulted in the specific knockdown of the corresponding gene, and this dsRNA-mediated silencing resulted in a loss of a function in the adult phenotype. All adults that appeared were mobile and had well-differentiated adult-like appendages, but some beetles retained an unhardened and untanned stratum corneum and incompletely spread wings. Based on the present results, the hypothetical effects of *DaAST* on JH biosynthesis in the pupal stage of *D. armandi* can be speculated. Treatment of early pupae with the JH mimics methoxypentadiene or hydropentadiene, resulting in the formation of a second pupal cuticle during adult molting [55,80]. The phenotype is similar to that processed by dsAST-treated phenotypes. Knockout of *AST* alone resulted in a significant increase in pupae mortality, a significant decrease in cumulative emergence and a significant malformation in emergent adults. After knockdown of *DaJHAMT* alone, the mortality rate of pupae increased, the cumulative emergence rate decreased significantly, and the emergence malformation rate did not change significantly. These results indicate that *DaAST* inhibits JH biosynthesis in *D. armandi*. However, these results did not contribute to determining whether *DaAST* regulates JH biosynthesis in larvae, as *DaAST* is not only expressed in the brain; the increased mortality could be due to something other than JH or *JHAMT*. In male adults, knockdown of *DaJHAMT* but not *DaAST* led to a dramatic decrease in frontalin synthesis. This suggests that in adult males, *DaAST* does not directly inhibit JH synthesis, since the negative effects of *DaAST* gene silencing in *D. armandi* may also be caused by their myoregulatory properties and other putative functions [32,81,82]. 

## 4. Materials and Methods

### 4.1. Insects 

We collected *Pinus armandii* Franch infested with *D. armandi* on the southern slopes of the central Qinling Mountains (33°18′–33°28′ N, 108°21′–108°39′ E) in Shaanxi, China, and placed the specimens in a greenhouse. The adult insects were collected after they emerged and were stored on moist paper at 4 °C. The sex of adults was based on external genitalia and male-specific auditory cues [83,84]. Larvae and pupae were collected from under the bark of infected *P. armandii*. 

### 4.2. RNA Isolation and cDNA Synthesis

Total RNA was isolated from three beetles by the UNIQ-10 Column Trizol Total RNA Isolation Kit (Sangon Biotech, Shanghai, China) in accordance with the manufacturer’s protocol. Its integrity was checked on 1% agarose gels and quantified using NANO DROP 2000 spectrophotometry (Thermo Scientific, Pittsburgh, PA, USA). The purity was calculated by means of the A260/A280 ratio (μg/mL = A260 × dilution factor × 40). The synthesized cDNA obtained from the sample was used as the template with the TransScript One-Step gDNA Removal and cDNA Synthesis SuperMix (TransGen Biotech, Beijing, China).

### 4.3. Amplification of Genes, Cloning and Sequence Analyses

cDNA synthesized from the sample was used as a template for the PCR reaction. Specific primers (Table S1) were designed in Primer Premier 5.0, based on the *AST* and *JHAMT* sequences of *D. ponderosae* from NCBI (http://www.ncbi.nlm.nih.gov/, accessed on 12 February 2021). PCR amplifications were performed in a C1000 thermocycler (Bio-Rad, Hercules, CA, USA), and the cDNA amplification was carried out in a 20 μL reaction volume: 1 μL cDNA, 0.25 μM of each primer, and 10 μL EcoTaq PCR SuperMix (TransGen Biotech, Beijing, China), with ddH_2_O added to 20 μL. The reaction conditions were as follows: 94 °C for 5 min, 30 cycles of 94 °C for 30 s, TM of each pair of primers for 30 s and 72 °C for 30 s, with a final extension for 10 min at 72 °C. The PCR products were visualized on 1% agarose gels stained with 1× DuRed and compared with a 2 K plus DNA marker (TransGene Biotech, Beijing, China). 

Single-stranded 5′and 3′ RACE-ready cDNA was synthesized from RNA using a SMARTer RACE cDNA Amplification Kit (Clontech Laboratories Inc., Mountain, CA, USA) according to the manufacturer’s protocol. Partial sequences were used in the primer design, and the PCR was performed as described in the SMARTer™ RACE cDNA Amplification Kit (Clontech Laboratories Inc., Mountain, CA, USA). The amplicons were purified, cloned and sequenced. Sequences were manually edited with EditSeq from DNASTAR (https://www.dnastar.com/, accessed on 6 March 2021) to obtain inserts, which were then BLASTed against the NCBI database. The complete sequences were compared using a BlastP search with those deposited in GenBank [62]. 

### 4.4. Sequence Analyses of the Genes

The molecular mass (kDa) and the isoelectric point (IP) of the two sequences were determined by the ProtParam program [63]. *DaAST* and *DaJHAMT* of *D. armandi* were checked for likely subcellular localization using Target P1.1 software (http://www.cbs.dtu.dk/services/TargetP/, accessed on 21 March 2021) with the default parameters [64].

In order to identify *AST* and *JHAMT* in *D. armandi*, a phylogenetic inference analysis of 11 full-length sequences was performed by the neighbor-joining method with MEGA7.0 [85,86]. To estimate the support for each node, bootstrap values were calculated after 1000 pseudoreplicates.

### 4.5. Analysis of the DaAST and DaJHAMT Genes Transcript Levels (Real Time-qPCR)

#### 4.5.1. Expression Patterns of Different Life Stages and Tissues

During development, *D. armandi* larvae were separated into three sub-stages: small larvae (SL: earlier instar larva whose weight is less than 2.5 mg); large larva (LL: large one is final instar larva but still feeding and the weight of the larva is between 5.0 and 7.0 mg); mature larvae (ML: when they stopped feeding in preparation for pupation). Pupae were separated into five sub-stages: P0: pupae, P1: Day 1 of the pupal stage, P2: Day 2 of the pupal stage, P3: Day 3 of the pupal stage and P4: Day 4 of the pupal stage. *D. armandi* adults were separated into four sub-stages: teneral adults (TA: body color still light), dark brown adults (DbA: decayed feather hole, body color was dark brown), emergent adults (EA), and feeding adults (invading a new host) There were three biological replicates per developmental stage, each containing three insects [83]. 

In terms of tissue distribution, for tissue-specific analysis of *DaAST* and *DaJHAMT* genes, 60 males and 60 females at the emergent adult stage (head, anterior midgut, hindgut, Malpighian tubule, fat body, reproductive organ (testes of males and ovaries of females) and antennae), 30 larvae and 30 pupae (head, gut, fat body, epidermis) were dissected, frozen immediately in liquid nitrogen and stored at −80 °C. Each tissue was replicated three times, and a pool of total RNA extracted from different tissues was used per replicate. RNA isolation and cDNA synthesis followed the protocols described above.

#### 4.5.2. Effects of JHA Injection on Transcript Levels of *DaAST* and *DaJHAMT*

Solutions of the original juvenile hormone analog JHA methoprene (Sigma, Saint Louis, MO, USA), were separately diluted to 5, 25 and 100 μg/μL concentrations using acetone [87]. Next, 0.1 μL of each JHA dilution was injected into *D. armandi* larvae and pupae through the ventral abdomen using Hamilton Microliter syringes (700 series, RN) with 32G sharp-point needles (Hamilton, Switzerland) to a final JHA content of 0.5, 2.5 or 10 μg. Meanwhile, an equal amount of acetone was injected as the solvent control. To analyze the expression of the JH-induced genes, the total RNA was extracted after 0, 24, 48 and 72 h of JHA or acetone treatment and subjected to cDNA synthesis and qRT-PCR.

### 4.6. dsRNA Synthesis 

#### 4.6.1. Target Genes

The *AST* and *JHAMT* genes of *D. armandi* were identified in a previous clone. Partial sequences of the *DaAST* and *DaJHAMT* genes (GenBank Accession No: MW645339 and MW645341) were appended with *Not*I and *Xba*I. The *DaAST* and *DaJHAMT* sequences were amplified with primers (Table S1) using EcoTaq PCR SuperMix (TransGen Biotech, Beijing, China) and a C1000 thermo cycler (Bio-Rad, Hercules, CA, USA). The polymerase chain reaction (PCR) amplification reaction conditions were as mentioned earlier. 

#### 4.6.2. Vector Construction and Expression

1. Construction of transformed *E. coli* expressing dsRNA

PCR products obtained in the previous steps were excised and cloned into the plasmid vector L4440 (Wuhan Miaoling Biotechnology Co., Ltd., Wuhan, China), between the *Not*I and *Xba*I restriction sites. Successful cloning was verified through PCR and sequencing. Plasmids containing the correct insert were extracted and transformed into *E. coli* strain HT115 (DE3) (Shanghai Weidi Biotechnology Co., Ltd., Shanghai, China). Positive clones were incubated at 37 °C until the mid-exponential phase (OD600 = 0.4). To activate the T7 promoter for RNA transcription, IPTG (isopropyl-β-D-1-thiogalactopyranoside) was added to a final concentration of 0.8 mM and then incubated for an additional 4 h under the same conditions. Each bacterial culture (100 mL) was transferred into a 50-mL Falcon tube and centrifuged at 4000× *g* for 10 min at 4 °C.

2. Isolation of dsRNA using conventional method

Cells were harvested via centrifugation at 5000× *g* and 4 °C for 10 min. DsRNA was isolated from 1 mL of the cell suspension with the UNIQ-10 Column Trizol Total RNA Isolation Kit (Sangon Biotech, Shanghai, China) according to the manufacturer’s protocol. The extracted RNA was compared with the dsRNA not induced by IPTG to determine whether IPTG had been successfully induced. Its integrity was checked on 1% agarose gels, and quantification was performed by spectrophotometry with a NANO DROP 2000 (Thermo Scientific, Pittsburgh, PA, USA). The reactions were allowed to proceed overnight at 42 °C, followed by both the RNase and DNase digestion and purification steps to obtain the dsRNA. The dsRNA was spectrophotometrically quantified before injection

Based on the L4440 vector, two expression vectors, L4440-AST and L4440-JHAMT, corresponding to *DaAST* and *DaJHAMT*, were constructed. The plasmids were digested by the restriction endonuclease *Not*I and *Xba*I. The gel electrophoresis results indicated that there were two bands; one is about 390 bp from L4440-AST, and the other 570 bp from L4440-JHAMT. After HT115 harbouring the plasmids, L4440-AST or L4440-JHAMT was induced by IPTG; we extracted the total RNA (containing dsAST or dsJHAMT) from the engineered bacteria (Figure S1). The gel electrophoresis results showed that the remainder was the band of dsAST and dsJHAMT (Figure S1).

#### 4.6.3. RNAi Experiment 

Larvae and pupae were collected under the bark of infected *P. armandii*. Male and female beetles were sexed upon emergence on the basis of external genitalia and male-specific auditory cues [83,84] and used in experiments immediately after sexing. For RNAi exposure, synthesized dsRNA (0.2 μL, concentration: 1000 ng/μL) was injected into the ventral abdomen of the larvae on the first day of the last instar or of pupae using a 10 μL Hamilton Microliter syringes (700 series, RN) with 32G sharp-point needles (Hamilton, Switzerland). Individual adult beetles (N = 25) were injected with two separate dsRNA treatments (*AST* or *JHAMT*) of 0.2 μL (concentration: 1000 ng/μL). Following dsRNA ingestion, beetles from each treatment were placed together in petri dishes containing damp filter paper; dishes were oriented vertically and maintained at 23 °C in the dark. Beetles were evaluated after 24 h. DEPC-treated water was used as a negative control. Untreated beetles were used as blank controls. Each beetle was injected only once. For each dose, three of the treated beetles were randomly selected at 24 h and 72 h, frozen immediately in liquid nitrogen and stored at −80 °C for further experiments. The expression levels of *DaAST* were quantified first, and the expression of *DaJHAMT* was quantified only in beetles in which *AST* was successfully knocked down. Twenty-five larvae were observed and the survival rate was recorded, and 25 pupae were observed for defective wings after plumentation and repeated three times. 

#### 4.6.4. RNA Interference and Quantification of Frontalin

In order to understand the effects of *DaAST* and *DaJHAMT* on the pheromone biosynthesis of male *D. armandi*, *D. armandi* males were injected with 200 ng of dsRNA (concentration: 1000 ng/μL) through the ventral abdomen using Hamilton Microliter syringes (700 series, RN) with 32G sharp-point needles (Hamilton, Switzerland). Beetles were treated according to the method of Sun et al. (2021) [88]; GC-MS determination also followed the methodology of Sun et al. to detect the content of frontalin [84,88,89].

### 4.7. Real-Time PCR

Specific qRT-PCR primers were designed by Primer Premier 5.0 on the basis of the obtained nucleotide sequences (Table S1). The melting curve analysis was performed to ensure that only a single product corresponding to the target sequence was amplified. All primer pairs were tested in advance to obtain close to 100%. The expression of the *CYP4G55* [90] and *β-actin* [83] genes was used as an internal control. Real-time PCR was performed in triplicate according to the manufacturer’s instructions using TransStart Top Green qPCR SuperMix (TransGen Biotech, Beijing, China) on a CFX96TM Real-Time qPCR Detection System (Bio-Rad, Hercules, CA, USA). The qPCR was performed using the following program: 95 °C for 10 min; 40 cycles at 95 °C for 5 s, TM of each pair of primers (Table S1) for 15 s and 72 °C for 20 s.

### 4.8. Statistics 

The 2^−ΔΔCt^ method was used to determine the effect of interference. According to this rule of thumb, transcript levels lower than 0.5 was considered to show that the effect of RNAi was significant. One-way analysis of variance (ANOVA) (*p* < 0.05) and two-way analysis was used to determine the significance of different treatments, and Tukey’s test was used to identify differences among treatments. For the gene silencing analysis, an unpaired *t*-test was used to compare differences in JH biosynthesis genes. The Kaplan–Meier method (log rank (Mantel–Cox)) was used to analyze the survival rates (*p* < 0.05) [91]. All statistical analyses were performed using SPSS Statistics 21.0 (IBM, Chicago, IL, USA) and plotted using Prism 5.0 (GraphPad Software, San Diego, CA, USA).

## 5. Conclusions

In general, injection of dsAST significantly increased the expression of *DaJHAMT* in larvae and adults, but had no significant effect on the pupae. Injection of dsAST and dsJHAMT had no significant effect on larval survival. Injection of dsAST and dsJHAMT into pupae resulted in decreased emergence rates, but dsAST resulted in approximately 50% greater susceptibility to deformation than dsJHAMT. Knockdown of *DaAST* seemed to enhance JH biosynthesis in pupae. In male adults, the intake of dsJHAMT reduced frontalin synthesis to zero, but injection of dsAST had no effect on frontalin synthesis. 

## Figures and Tables

**Table 1:** Amino acid identity of putative allatostatin (*DaAST*) and juvenile hormone acid O-methyltransferase (*DaJHAMT*) with related sequences in other insect species.

Genes	BLAST Matches in Genbank			Identity ^1^
	Species	Gene	Accession No.	BlastP
*DaAST*	*Dendroctonus ponderosae*	*AST*	XP_019760338.1	69%
	*Tribolium castaneum*	*AST*	NP_001137205.1	67%
	*Leptinotarsa decemlineata*	*AST*	AIW62334.1	64%
*DaJHAMT*	*Dendroctonus ponderosae*	*JHAMT*	XP_019760146.1	53%
	*Nicrophorus vespilloides*	*JHAMT*	XP_017772157.1	36.8%
	*Aethina tumida*	*JHAMT*	XP_019876726.1	36.5%

^1^ As predicted by BLAST (www.ncbi.nlm.nih.gov, accessed on 12 February 2021) [62].

**Table 2:** Physicochemical properties and cellular localization of *DaAST* and *DaJHAMT* of *D. armandi*.

Gene Name	Accession No.	Full Length (bp) ^1^	ORF Size (aa/bp) ^1^	Mw (kDa) ^1^	I.P. ^1^	Signal Peptide Prediction ^2^
*DaAST*	MW645339	471	114/345	13.28	8.66	SP 0.766 mTP 0.043 other 0.194
*DaJHAMT*	MW645341	1137	275/828	15.11	9.04	SP 0.069 mTP 0.062 other 0.932

^1^ As predicted by ProtParam [63]. ^2^ As predicted by Target P 1.1 [64]. I.P.: isoelectric point; Mw: molecular weight; ORF: open reading frame; SP: secretory pathway signal peptide; mTP: mitochondrial targeting peptide.

**Table 3:** Adult development of the dsRNA phenotypes.

Groups	Emergence (%)	Adult Deformity (%)	Death of Deformed Adults (%)	Days of Death (d)
Control	72.00	16.39	66.67	4
Non-injected	78.67	1.33	100	3
dsAST	52.00	54.46	71.42	1
dsJHAMT	52.00	15.45	100	2

## Data Availability

The datasets generated during and/or analyzed during the current study are available from the corresponding author on reasonable request.

## Supplementary Materials

The following supporting information can be downloaded at: https://www.mdpi.com/article/10.3390/ijms23052749/s1.
