# Supplementary material for: Functional Characterization of Allatostatin C (PISCF/AST) and Juvenile Hormone Acid O-Methyltransferase in Dendroctonus armandi
Source: Int J Mol Sci. 2022 Mar 2;23(5):2749. doi: 10.3390/ijms23052749 (PMC8910878; doi:10.3390/ijms23052749)
Supplement: Supplementary file 1 [file ijms-23-02749-s001.zip › ijms-1589968-supplementary.pdf]

Table S1. Primers designed by Primer Primier 5.0 used in the research.

Genes name		Primer direction (5'-3')	Application	TM (°C)	Amplification efficiency (%)	Product size (bp)
DaAST	*F*	ATGCTGCTCTATTCTTCAC	cDNA amplification			168
	R	CTTCGATGCCTCTAGTTAA				
DaJHAMT	*F*	ATTGGGTGGTGGAACAGAGGC	cDNA amplification			160
	R	CCGAATGGTGATAGGGCGAGA				
DaAST	*Inner*	TCAGCCAAATGAATAACAACCT	3' RACE			
	Outer	AGCATTGAGAAGTGGCAACGAA				
DaJHAMT	*Inner*	GCAAACAACCGACAATACC	3' RACE			
	Outer	GACCGAAATCAAAGTGCCT				
DaAST	*Inner*	TTTCGTTGCCACTTCTCAATGCTC	5' RACE			
	Outer	GGCCATTCTTTGGGCAAACAGCT				
DaJHAMT	*Inner*	GCACTTTGGGACGATTTCCTGACT	5' RACE			
	Outer	TCTTGATAATGAGGAGGTTT				
DaAST	*F*	CTCAGCCAAATGAATAA	RT-qPCR	54.3	102	119
	R	CACAGGGTTGAAGTAGC				
DaJHAMT	*F*	GGCTACACTACCGCCAACA	RT-qPCR	50	100.1	161
	R	GCAAGCGTCACTGTCCATC				
DaAACT	*F*	GTAAGTCTTGGTCACCCCATTGG	RT-qPCR	68	97.6	149
	R	CCAACTTACAATCGCTCCACCAT				
DaHMGR	*F*	GGATGGTCGTGAGTTGTA	RT-qPCR	56.6	94.6	187
	R	TTTGAAGTTATCTGGGTCT				
DaHMGS	*F*	CTACTTGGAGGCTCTGGAC	RT-qPCR	58.6	93.5	147
	R	CTTCTCCGAACCTGATTGC				
DaMK	*F*	AAGGCGACGCTGATATTGGTA	RT-qPCR	60	99.6	138
	R	ATCGTGAGATGATCGGTGGAG				
DaPMK	*F*	GGGTAGATGACGCTGAGTCCGAATG	RT-qPCR	69.5	103.9	147
	R	TGCACCGCCACCAAAGAAAACTGT				
DaMPDC	*F*	GCCACAAGAATTAGATCACCCAAG	RT-qPCR	63	92.2	158
	R	ACCCCAACCATCCAAAATACCACC				
DaIDI	*F*	TACAAGGACCAAGGAAACG	RT-qPCR	55.8	91.6	160
	R	AGTGGGAATAAAGTCGTCAA				
DaFPPS	*F*	AAACCACTTTGGGTCAGA	RT-qPCR	58.6	97.3	185
	R	TCGGTGTTGTTCCTCGTC				
CYP4G55	*F*	ATGGCTTTCTTGGATCTCCT	RT-qPCR	60	100.2	113
	R	GCAGTCGTATCGTGACCCTC				
β-actin	*F*	CATCAGGAAGGACTTGTA	RT-qPCR	60		124
	R	GATTCGTCGTATTCTTGTT				
DaAST	*F*	ATAAGAATGCGGCCGCAGGAAATCGTCCCAAAGT	dsRNA synthesis			390
	R	TGCTCTAGACACAGGGTTGAAGTAGCA				
DaJHAMT	*F*	ATAAGAATGCGGCCGCGGCTACACTACCGCCAACA	dsRNA synthesis			570
	R	TGCTCTAGATGCTCACCTCCTTCGTAAA				

Note: *Not*I and *Xba*I endonuclease sequences are underlined

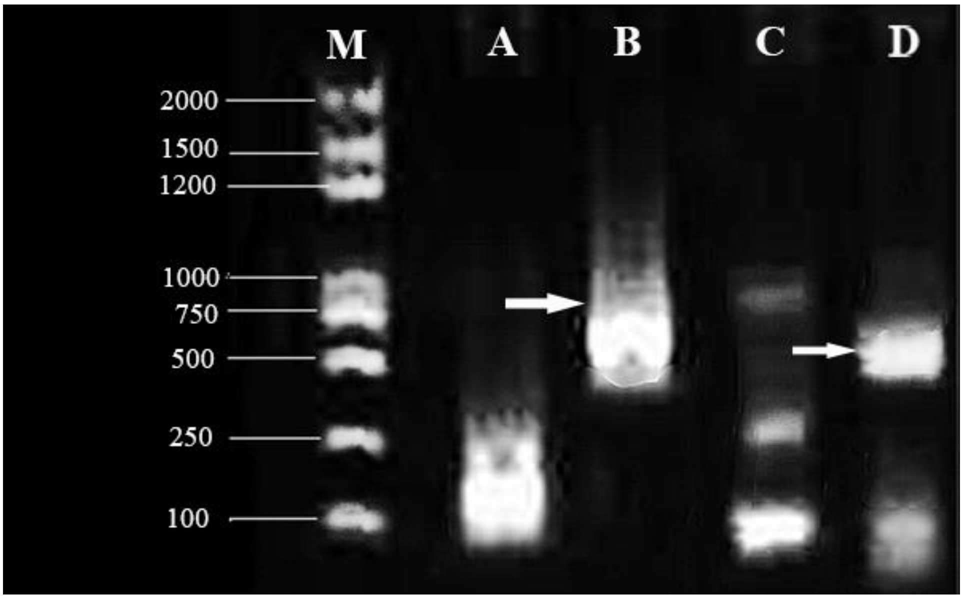

Figure S1. Confirmation of dsRNA produced in HT115 cells. The recombinant plasmids were transformed into HT115-competent cells. Individual transformants were cultured on 2 × YT media with the addition of IPTG (B: dsAST; D: dsJHAMT) or without the addition of IPTG (A, C). The cell cultures were processed for total RNA extraction. M, 2 kb plus DNA marker (TIANGEN, China) The arrowheads indicate the position of the dsRNA band.
